# Demographic and health-related factors associated with reduced work functioning in people with moderate medically unexplained physical symptoms: a cross-sectional study

**DOI:** 10.1186/s12889-020-09415-9

**Published:** 2020-08-31

**Authors:** Mark L. van Tilburg, Paula Elisabeth van Westrienen, Martijn F. Pisters

**Affiliations:** 1 Department of Rehabilitation, Physiotherapy Science and Sports, UMC Utrecht Brain Center, University Medical Center Utrecht, Utrecht University, Utrecht, the Netherlands; 2 Expertise Center Healthy Urban Living, Research Group Innovation of Human Movement Care, University of Applied Sciences Utrecht, Heidelberglaan 7, 3584 CS Utrecht, the Netherlands; 3 Center for Physical Therapy Research and Innovation in Primary Care, Julius Health Care Centers, Utrecht, the Netherlands; 4 Department of Health Innovation and Technology, Fontys University of Applied Sciences, Eindhoven, the Netherlands

**Keywords:** Absenteeism, Work performance, Medically unexplained symptoms

## Abstract

**Background:**

Medically unexplained physical symptoms (MUPS) are a leading cause of reduced work functioning. It is not known which factors are associated with reduced work functioning in people with moderate MUPS. Insight in these factors can contribute to prevention of reduced work functioning, associated work-related costs and in MUPS becoming chronic. Therefore, the aim of this study was to identify which demographic and health-related factors are associated with reduced work functioning, operationalized as impaired work performance and absenteeism, in people with moderate MUPS.

**Methods:**

Data of 104 participants from an ongoing study on people with moderate MUPS were used in this cross-sectional study. Ten independent variables were measured at baseline to determine their association with reduced work functioning: severity of psychosocial symptoms (four domains, measured with the Four-Dimensional Symptom Questionnaire), physical health (RAND 36-Item Health Survey), moderate or vigorous physical activity (Activ8 activity monitor), age, sex, education level and duration of complaints. Two separate multivariable linear regression analyses were performed with backward stepwise selection, for both impaired work performance and absenteeism.

**Results:**

Absenteeism rate rose with 2.5 and 0.6% for every increased point on the Four-Dimensional Symptom Questionnaire for domain ‘depression’ (*B* = 0.025, *SE* = 0.009, *p* = .006) and domain ‘somatization’ (*B* = 0.006, *SE* = 0.003, *p* = .086), respectively. An *R*^2^ value of 0.118 was found. Impaired work performance rate rose with 0.2 and 0.5% for every increased point on the Four-Dimensional Symptom Questionnaire for domain ‘distress’ (*B* = 0.002, *SE* = 0.001, *p* = .084) and domain ‘somatization’ (*B* = 0.005, *SE* = 0.001, *p* < .001), respectively. An *R*^2^ value of 0.252 was found.

**Conclusions:**

Severity of distress, probability of a depressive disorder and probability of somatization are positively associated with higher rates of reduced work functioning in people with moderate MUPS. To prevent long-term absenteeism and highly impaired work performance severity of psychosocial symptoms seem to play a significant role. However, because of the low percentage of explained variance, additional research is necessary to gain insight in other factors that might explain the variance in reduced work functioning even better.

## Background

Medically unexplained physical symptoms (MUPS) are a common phenomenon in primary care [1–3]. MUPS are defined as physical complaints that last for more than a few weeks and cannot be explained by a medical condition after a proper medical examination [4, 5]. Approximately, 25–50% of all symptoms for which people visit a health professional cannot be medically explained immediately [2]. These symptoms can be pain or fatigue and can affect any anatomical structure or body region [6]. MUPS can be categorized into mild MUPS (2 or less MUPS-related GP consults during the last 12 months), moderate MUPS (3 or more MUPS-related GP consults during the last 12 months) or chronic MUPS [7]. Although symptoms might reoccur, mild MUPS usually recover within a few weeks to three months [7]. Moderate MUPS have an estimated prevalence of approximately 15% [8]. People with moderate MUPS still experience symptoms after three months, without having a diagnosis of a persistent somatic symptom disorder or functional somatic syndrome [8]. Chronic MUPS are characterized by the presence of a somatic symptom disorder or functional somatic syndrome according to the Diagnostic and Statistical Manual of Mental Disorders, Fifth Edition (DSM-V), such as fibromyalgia, chronic fatigue syndrome or irritable bowel syndrome [9]. Only 2.5% of people with MUPS in primary care are identified with chronic MUPS [2]. Olde Hartman et al. showed in their systematic review that in the overall MUPS population symptoms improve in approximately 50% of people and that 10 to 30% of people with MUPS deteriorate [3].

The burden of chronic MUPS for patients, health professionals and society is high [2]. People with chronic MUPS experience persistent pain, fatigue, a decreased quality of life and feel more socially isolated [6, 10]. Health professionals struggle with the disease management of patients with chronic MUPS and the treatment requires substantial time commitment [11, 12]. This struggle with the disease management in combination with patients’ illness beliefs of having a progressive illness can lead to inappropriate use of healthcare resources [13]. People with chronic MUPS visit a medical specialist approximately 7 to 8 times and a health professional approximately 13 to 15 times per year. Often specialists or health professionals miss the MUPS diagnosis and want patients to receive somatic interventions, or have fear to miss out on a medical diagnosis. These visits can be ineffective or even iatrogenic, because they might attribute to patients’ beliefs of needing biomedical instead of bio-psycho-social interventions [14–17]. The inappropriate use of healthcare resources and medication also leads to substantial excessive direct costs in health care [14, 15, 18]. All subgroups of MUPS are not only associated with direct costs, but also with high indirect costs. A substantial part of indirect costs is work-related [15, 18]. Roelen et al. reported a high prevalence of MUPS (78%) among Dutch working personnel in a library, administrative office, a cheese factory and a metal producing company [19]. Hiller et al. estimated that indirect work-related costs are three times as high as direct costs in people with chronic MUPS [20]. Work-related costs can be explained by the fact that MUPS lead to reduced work functioning, in terms of absenteeism or impaired work performance due to presenteeism [21]. Absenteeism is operationalized as the workdays a person was absent divided by the workdays a person was supposed to work in the last four weeks [22, 23]. Impaired work performance due to presenteeism (productivity loss due to working while sick or experiencing MUPS) is operationalized as the amount of lost working hours per week due to health-related productivity loss [22, 23]. Over 80% of the people with MUPS, with no or only partial absenteeism, reported impaired work performance [14]. Zonneveld et al. reported in their study that impaired work performance in people with MUPS costs €855.79 per employed person per year (PPPY) [14]. These costs are based on two lost working hours per workweek. Furthermore, absenteeism in people with MUPS costs by estimate €2403.92 PPPY. These costs are based on the estimate of 67 disability days per employed person per year [14]. Löwe et al. reported comparable results of 18.2 disability days per person per three months in people with MUPS [24]. Although these numbers are related to all subgroups of MUPS, Rask et al. concluded that not only chronic MUPS have significant impact on work functioning, but also mild and moderate MUPS [21].

Despite MUPS being a leading cause of absenteeism, a systematic review by Aamland et al. identified only a small number of studies concerning factors associated with absenteeism in the overall MUPS population [25]. Additionally, there is a lack of knowledge about people with moderate MUPS in general. After critical appraisal of existing literature in patient groups that might be comparable to the MUPS population, several factors hypothesized to be associated with reduced work functioning in people with moderate MUPS were identified. Roelen et al. defined an association between psychiatric comorbidity as well as severity of symptoms and absenteeism [19]. Compare et al. found in a working population of people with non-specific low back pain that sex, type of work and physical behaviour were significantly associated with reduced work functioning [26]. In a qualitative study in people with spine related pain (SRP), duration of complaints was mentioned as an important factor related to absenteeism. People who were previously unfamiliar with SRP prioritized their SRP over work and reported sick sooner than people that had endured episodes of SRP before [27]. Knowledge about these factors in the moderate MUPS population can help to understand why the degree of reduced work functioning varies from person to person for apparently comparable symptom burdens. Therefore, the aim of this study was to identify which demographic and health-related factors are associated with reduced work functioning, operationalized as impaired work performance and absenteeism, in people with moderate MUPS.

## Methods

### Study design

Which demographic and health-related factors are associated with reduced work functioning, operationalized as impaired work performance and absenteeism, in people with moderate MUPS was studied with a cross-sectional multivariable modelling study design.

### Setting and characteristics of participants

Data used in this cross-sectional study were collected as part of a large study on a blended and integrated mental health nurse and physical therapy intervention program (PARASOL) [28]. In the PARASOL study, adult patients with moderate MUPS were i.a. recruited by their general practitioner (GP) from 14 Dutch primary health care centres (Leidsche Rijn Julius Health Care Centres and the Eindhoven Corporation of Primary Health care Centres), using the preventive screening of medically unexplained physical symptoms (PRESUME) screening method [7]. In the first two steps of the PRESUME screening method, patients of 18 years or older who have had at least five general practice consultations during the past 12 months were selected and excluded by their GP if they: received a MUPS targeted multidisciplinary intervention in the past 12 months, received a medically explained diagnosis between identification and the time of inclusion or were unable to participate according to the GP, for instance because of a life-threatening condition, a shortened life expectancy or a major life event in the past month. In the third step of the PRESUME screening method, patients were assigned to the moderate MUPS subgroup if they: have had three or more contacts with the GP with one of the 104 ICPC codes suggestive of MUPS, as assessed by the GPs during regular care (symptom diagnoses) [7]. At last, patients were excluded if they had insufficient mastery of the Dutch language or had no access to the internet [28]. In the current cross-sectional study on impaired work performance and absenteeism, all patients who had a job in the four weeks prior to the baseline measurements were included. Participant inclusion lasted from March 2017 until April 2018 [28].

### Data collection

#### Dependent variables

The main dependent variable in this cross-sectional study was work functioning. Work functioning was operationalized as two variables: absenteeism and impaired work performance. Both variables were assessed with the Trimbos/Institute for Medical Technology Assessment Questionnaire for costs associated with Psychiatric Illnesses (TiC-P). The TiC-P is a feasible and reliable instrument for collecting data on medical consumption and productivity loss in people with mental health problems [29]. The recall-period of the TiC-P is 4 weeks.

#### Absenteeism

Absenteeism rates were computed by dividing the number of days absent from work due to health problems during the last four weeks by the number of workdays a person was supposed to work in the last four weeks. A higher rate indicates more absenteeism in the last 4 weeks [22, 23].

#### Impaired work performance

Impaired work performance rates were computed by a formula based on two items of the TiC-P. The first question was: “On how many workdays during the last 4 weeks did you perform paid work, although you were bothered by health problems?” The second question was: “Please rate how well you performed on the days you went to work even though you were suffering from health problems” which the respondent rated on a 10-point scale (0.0 = maximally inefficient, 1.0 = efficient as usual) [22, 23]. A higher rate indicates more impaired work performance [23]:

$$ \mathrm{impaired}\ \mathrm{work}\ \mathrm{performance}=\frac{\mathrm{days}\ \mathrm{hindered}\ast \left(1-\mathrm{efficiency}\right)\ast \mathrm{work}\ \mathrm{hours}\ \mathrm{per}\ \mathrm{day}}{\mathrm{work}\ \mathrm{hours}\ \mathrm{per}\ \mathrm{week}} $$

### Independent variables

Ten independent variables were measured to determine which of the factors were associated with reduced work functioning. Measured independent variables were: severity of psychosocial symptoms (four domains), physical health, moderate or vigorous physical activity, age, sex, education level and duration of complaints. These variables were selected following a review of the literature and subsequently the consensus opinion by the research group [19, 26, 27].

Severity of psychosocial symptoms was measured with the Four-Dimensional Symptom Questionnaire (4DSQ) [30, 31]. The 4DSQ is a valid questionnaire, which measures 4 domains: distress (0–32), depression (0–12), anxiety (0–24), and somatization (0–32). Each domain consists of multiple items. After scoring the items, the sum of all items for each domain was calculated. A higher score defines an increased probability of a disorder [30, 31].

Physical health was measured with the RAND 36-Item Health Survey (RAND-36) [32, 33]. The RAND-36 is a valid and reliable questionnaire, which measures eight domains: physical functioning, vitality, emotional well-being, social functioning, pain, general health, role limitations due to physical health, and role limitations due to emotional problems. Because the 4DSQ already covered the mental aspect in the analyses, only the physical health component summary score (PCS) of the RAND-36 was used in the analyses [34, 35]. A score above 50 means a more favourable physical health state compared to the Dutch reference population [32, 33, 36].

Physical activity was measured with the Activ8 activity monitor. The Activ8 is a valid instrument to quantify movement and motion [37]. Participants wore the Activ8 in their trouser pocket or in a leg strap for one week. Data were transformed into the average amount of hours of moderate or vigorous physical activity per day (MVPA) [37]. Physical activity with a metabolic equivalent of ≥3, was considered MVPA [38].

Age, sex (male; female), education level (higher general/preparatory academic education or lower; higher professional education or higher) and duration of complaints (< 2.5 years; ≥2.5 years) were assessed with a self-administered questionnaire. A cut-off point of 2.5 years for duration of complaints was chosen, because people with MUPS often experience symptoms for a long time, before they are diagnosed with MUPS [39].

### Statistical analysis

Statistical analyses were performed with IBM SPSS statistics for Windows (version 24, IBM corp. Armonk, NY, USA). If missing data were less than 10% and under the missing (completely) at random assumption, missing values of independent variables were imputed with ‘Multivariate Imputation by Chained Equations’ [40]. Analyses were performed with the pooled imputed data. The variance inflation factor (VIF) was calculated to assess multicollinearity. A VIF value greater than 5 represents a critical level of multicollinearity [41]. In the impaired work performance analysis, outliers of more than three standard deviations from the mean were discarded, because a sensitivity analysis showed that these affected the regression model significantly.

Two separate univariable and two separate multivariable linear regression analyses were performed (impaired work performance and absenteeism) to assess the association of the chosen independent variables with work functioning. Linear multiple regression analyses with backward stepwise selection were performed, after assumptions for linear regression were checked. Linearity, homogeneity of residuals and the assumption of normality were tested with plots of residuals, and the assumptions of linearity and normal distribution were checked [42–45]. A *p* value of > 0.1 was chosen for removal of variables. For each variable, unstandardized regression coefficients (*B*) with a standard error (*SE*) were calculated.

The *R*^2^ statistic was calculated to assess the overall performance of the final model in predicting the degree of reduced work functioning. Means and standard deviations (continuous) or percentages (categorical) of both the dependent and independent variables were calculated by using descriptive statistics.

## Results

A total of 104 participants were included in the present study. Descriptive characteristics of the study population are presented in Table 1. Participants had a less favourable physical health state compared to the Dutch reference population (*M =* 42.9*, SD* = 5.2) [32, 33, 36]. Additionally, severity of psychosocial symptom scores were mildly elevated for domains distress (*M =* 11.8, *SD =* 7.7) and somatization (*M =* 12.7, *SD* = 6.8) and were low for depression (*M* = 1.4, *SD =* 2.5) and anxiety (*M* = 2.5, *SD* = 3.6). Some rate of absenteeism was reported in 33% of the participants. Also, impaired work performance was reported in 57% of the 104 participants. All variables had less than 10% missing values and were under the missing (completely) at random assumption. Missing data were imputed and analyses were performed with the pooled imputed data.

**Table 1:** Descriptive characteristics of study population, including all studied variables (*N* = 104)

Variable	Value
**Age**	43.9 ± 10.7
**Sex**, female (%)	73.1
**Education level**, high (%)	40.4
**Duration of physical complaints**, ≥2.5 years (%)	65.4
**Physical health (RAND-36; PCS)**	42.9 ± 5.2
**Severity of psychosocial symptoms (4DSQ)**	
Distress	11.8 ± 7.7
Depression	1.4 ± 2.5
Anxiety	2.5 ± 3.6
Somatization	12.7 ± 6.8
**MVPA (average hours per day)** (median (IQR))	0.3 (0.6)
**Absenteeism**, yes (%)	32.5
**Impaired work performance**, yes (%)	56.7

Data are presented as mean ± standard deviation

*Abbreviations: RAND-36* RAND 36-item Health Survey, *PCS* Physical Component Summary, *4DSQ* Four-Dimensional Symptom Questionnaire, *MVPA* average hours per day of moderate or vigorous physical activity; IQR, Interquartile Range

### Absenteeism

To assess the association of variables with absenteeism, all 104 participants were analysed. The results of the univariable regression analysis are presented in Table 2. The results of the multivariable regression analysis are presented in Table 3. Absenteeism rate rose with 2.5 and 0.6% for every increased point on the 4DSQ for domain ‘depression’ (*B* = 0.025, *SE* = 0.009, *p* = .006) and domain ‘somatization’ (*B* = 0.006, *SE* = 0.003, *p* = .086), respectively.

### Impaired work performance

To assess the association of variables with work performance, 102 participants were analysed. The level of impaired work performance deviated more than three standard deviations from the mean in two participants and were therefore discarded from the analysis. The results of the univariable regression analysis are presented in Table 2. The results of the multivariable regression are presented in Table 3. Impaired work performance rate rose with 0.2 and 0.5% for every increased point on the 4DSQ for domain ‘distress’ (*B* = 0.002, *SE* = 0.001, *p* = .084) and domain ‘somatization’ (*B* = 0.005, *SE* = 0.001, *p* < .001), respectively.

**Table 2:** Univariable associations between reduced work functioning and patient characteristics

	Absenteeism (***n*** = 104)			Impaired work performance (***n*** = 102)		
	***B***	***SE B***	***p***	***B***	***SE B***	***p***
**Age**	0.001	0.002	.503	< 0.001	0.001	.894
**Sex, female**	−0.012	0.058	.834	−0.007	0.020	.740
**High education level**	0.024	0.051	.635	0.001	0.018	.964
**≥2.5 years of complaints**	−0.047	0.052	.368	−0.012	0.018	.514
**Physical health (RAND-36; PCS)**	−0.002	0.005	.638	−0.001	0.002	.618
**Average hours per day of moderate or vigorous physical activity**	−0.009	0.051	.855	−0.020	0.018	.256
**Severity of psychosocial symptoms (4DSQ)**						
Distress	0.005	0.005	.315	0.003	0.002	.080
Depression	0.022	0.013	.099	−0.005	0.005	.271
Anxiety	−0.012	0.009	.171	−0.001	0.003	.849
Somatization	0.008	0.004	.054	0.005	0.002	< .001

*B*, unstandardized regression coefficient, *SE* Standard Error of the estimate, *RAND-36* RAND 36-item Health Survey, *PCS* Physical Component Summary score, *4DSQ* Four-Dimensional Symptom Questionnaire

**Table 3:** Multivariable associations between reduced work functioning and patient characteristics

	Absenteeism (n = 104)			Impaired work performance (n = 102)		
	***B***	***SE B***	***p***	***B***	***SE B***	***p***
**Constant**	0.002	0.046	.973	−0.007	0.017	.687
**Age**	e			e		
**Sex, female**	e			e		
**High education level**	e			e		
**≥2.5 years of complaints**	e			e		
**Physical health (RAND-36; PCS)**	e			e		
**Average hours per day of moderate or vigorous physical activity**	e			e		
**Severity of psychosocial symptoms (4DSQ)**						
Distress	e			0.002	0.001	.084
Depression	0.025	0.009	.006	e		
Anxiety	e			e		
Somatization	0.006	0.003	.086	0.005	0.001	< .001
***R***^**2**^ **statistic**	0.118		.002	0.252		< .001

*B*, unstandardized regression coefficient, *SE* Standard Error of the estimate, *e* variable excluded from the regression model, *RAND-36* RAND 36-item Health Survey, *PCS* Physical Component Summary score, *4DSQ* Four-Dimensional Symptom Questionnaire

## Discussion

In this cross-sectional study, the association between work functioning, operationalized as absenteeism and impaired work performance, and demographic and health-related factors was examined in people with moderate MUPS. We found that probability of a depressive disorder and probability of somatization were positively associated with a higher absenteeism rate. Furthermore, an increased severity of distress and probability of somatization were positively associated with a higher impaired work performance rate. The focus of this study was to gain insight in demographic and health-related factors associated with reduced work functioning. However, because of the low *R*^2^ value, it is likely that the total variance in reduced work functioning might be explained even better by factors that were not measured in the present study.

This study is one of the first to report insights in the moderate MUPS population. In the present study, the period prevalence of absenteeism over four weeks in people with moderate MUPS was 32.5% and the period prevalence of impaired work performance over four weeks was 56.7%. Zonneveld et al. reported in the overall MUPS population a period prevalence of absenteeism of 46.6% over two weeks and a period prevalence of impaired work performance of 85.4% over two weeks [14]. Period prevalence of absenteeism in the general Dutch working population was 45% over the full year 2016, but people reported sick for just 3.8% of the total time they were supposed to work [46]. These numbers are hard to compare because they are prevalence percentages for different time periods. However, our findings suggest that people with moderate MUPS are slightly less absent from work and substantially less impaired in their work performance compared to the overall MUPS population and probably have a substantially higher degree of reduced work functioning compared to the general Dutch working population.

Severity of psychosocial symptoms seems to play an important role in addressing reduced work functioning in people with moderate MUPS. Concerning the finding that an increased probability of a depressive disorder was associated with a higher absenteeism rate in the present study, it may be possible that an increased probability of a depressive disorder will lead to a higher absenteeism rate in people with moderate MUPS, since having a depressive disorder was shown to cause absenteeism in previous research [23]. Regarding the findings about an increased probability of somatization in the present study, it may be possible that an increased probability of somatization will lead to absenteeism and impaired work performance in people with moderate MUPS. This is supported by the results of Den Boeft et al. who reported that moderate or high risk of somatization is associated with a higher absenteeism rate, even after adjusting for depressive and anxiety disorders and job characteristics [22]. Concerning the finding that an increased probability of an increased severity of distress was associated with a higher impaired work performance rate, it may be possible that an increased severity of distress will lead to a higher impaired work performance rate in people with moderate MUPS, since psychological distress led to an increased risk of impaired work performance for a range of other health conditions in previous research [47]. Additionally, the low percentage of explained variance by the final models in the present study coincided with a study of Rask et al. who reported for the overall MUPS population that depressive and anxiety disorders influenced the association between MUPS and work functioning, but did not fully explain the effect [21]. Furthermore, no significant associations were found between demographic factors, duration of complaints, physical health, physical activity and reduced work functioning. This suggests that other factors might influence work functioning even more, such as a higher physically or emotionally demanding job, low support by colleagues, low task control and longer working hours, which were associated with reduced work functioning in the general population [22, 48–51]. Unfortunately, these factors were not measured in the PARASOL study and therefore could not be included in the analyses.

A strength of this cross-sectional study was that only the moderate MUPS population was included, by using the PRESUME screening method [7]. Since very little is known about this specific population, new insights in the moderate MUPS working population are valuable. However, it is unclear if the results only apply to the moderate MUPS working population, or reflect general observations that would be true in any other population. Another strength was that not only absenteeism was included as main outcome, but also impaired work performance due to presenteeism, since health-related productivity loss significantly contributes to work-related costs [21]. Nevertheless, this study had some limitations. The absenteeism analysis violated assumptions for linear regression. This caused the regression coefficients to be less reliable, and the confidence intervals to be rather large. A possible explanation for the violation of assumptions is that more than two-third of the participants reported no absenteeism. Therefore, the mean was low and relatively high values tended to be outliers. Additionally, a widely adopted rule of thumb to achieve an adequate power, is to have at least ten outcome events per independent variable, thus the sample size in this study may be inadequate [45]. However, since results of the present study coincided with research on reduced work functioning in other populations, results of the present study seem reliable [21–23, 47]. The impaired work performance analysis met the assumptions for linear regression. However, to meet these assumptions, two outliers had to be excluded from the analysis, leading to a relatively narrow range in impaired work performance in our sample. Other limitations of the present study were that reasons for absenteeism were not identified and impaired work performance was based on the number of days hindered by health problems, but not specifically by MUPS-related health problems. Other diagnosed health concerns might have caused additional impairment. This may have led to an overestimation of reduced work functioning rates. However, since reduced work functioning rates in our sample were substantially higher than the overall MUPS and the general Dutch population these findings are not likely to be overestimated [14, 46].

## Conclusions

Severity of distress, probability of a depressive disorder and probability of somatization are positively associated with higher rates of reduced work functioning in people with moderate MUPS. To prevent long-term absenteeism and highly impaired work performance, severity of psychosocial symptoms seem to play a significant role. However, because of the low explained variance, we suggest that future research should focus on the association or causality between severity of psychosocial symptoms, unfavourable job characteristics and reduced work functioning in people with moderate MUPS. Additionally, we suggest that future research should check if these results specifically apply to the moderate MUPS working population, or also apply to the overall MUPS population.

## Data Availability

The datasets generated during the PARASOL study are not publicly available, but datasets used and/or analysed during the current study are available from the corresponding author on reasonable request given appropriate ethical approvals are obtained.

## Abbreviations

4DSQ: Four-Dimensional Symptom Questionnaire
DSM-V: Diagnostic and Statistical Manual of Mental Disorders, Fifth Edition
GP: General Practitioner
MUPS: Medically Unexplained Physical Symptoms
MVPA: amount of hours of Moderate or Vigorous Physical Activity per day
PARASOL: (Dutch) ProActief zoRgprogrammA ter preventie van chroniciteit bij Somatisch Onvoldoende verklaarde Lichamelijke klachten
PCS: Physical health Component Summary core
PPPY: Per employed Person Per Year
PRESUME: preventive screening of medically unexplained physical symptoms
RAND-36: RAND 36-Item Health Survey
TiC-P: Trimbos/Institute for Medical Technology Assessment Questionnaire for costs associated with Psychiatric Illnesses
VIF: Variance of Inflation Factor
